# Unmasking the Culprits: A Case of Epstein-Barr Virus-Associated Hemophagocytic Lymphohistiocytosis Presenting With Mouth Ulcers and Nosebleeds

**DOI:** 10.7759/cureus.61822

**Published:** 2024-06-06

**Authors:** Ahmad Obeidat, Feras Al-Moussally, Daoud O Al Aruri, Esraa ALhomaimat, Kamyar Nader

**Affiliations:** 1 Department of Internal Medicine, MedStar Washington Hospital Center, Washington, DC, USA; 2 Department of Internal Medicine, University of Central Florida College of Medicine, Orlando, USA; 3 Department of Internal Medicine, Jordan University Hospital, Amman, JOR; 4 Department of Internal Medicine, Jordan University of Science and Technology, Irbid, JOR; 5 Department of Hematology and Oncology, MedStar Washington Hospital Center, Washington, DC, USA

**Keywords:** soluble interleukin-2 receptor, viremia, infection, immunocompetent adult, epistaxis, secondary hemophagocytic lymphohistiocytosis (hlh), mouth ulcer, ebv hlh, hemophagocytic lymphohistiocytosis (hlh)

## Abstract

Hemophagocytic lymphohistiocytosis (HLH) is an aggressive syndrome of excessive immune activation. It usually occurs in children, mainly during the first year of life. Primary hemophagocytic lymphohistiocytosis is more common and usually occurs in immunocompromised patients. Secondary hemophagocytic lymphohistiocytosis, on the other hand, is less common, especially in immunocompetent patients. Here, we intend to present a case of a 55-year-old male patient who had no known immune deficiency, presented with epistaxis, and was found to have Epstein-Barr virus (EBV)-induced hemophagocytic lymphohistiocytosis.

## Introduction

Hemophagocytic lymphohistiocytosis (HLH) is a life-threatening condition with a mortality rate reaching as high as 40% in adults [1]. Primary HLH occurs in the setting of a genetic mutation, while secondary HLH occurs secondary to a trigger such as an autoimmune process, malignancy, or, as in our case, an infection [1]. Most patients with HLH are acutely ill with multiorgan involvement [1]. Common findings include fever, hepatosplenomegaly, rash, lymphadenopathy, neurologic symptoms, pancytopenia, high serum ferritin, and liver function abnormalities [2]. This adds to the peculiarity of our case since our patient initially presented with only epistaxis and mouth ulcers.

## Case presentation

A 55-year-old male with a past medical history of chronic rhinosinusitis and right pre-orbital cellulitis presented with foul-smelling drainage and worsening epistaxis from the right nostril for two months. Of note, the patient was also complaining of mouth ulcers. His laboratory investigation on presentation was only significant for mild pancytopenia (Table 1).

**Table 1:** The patient’s laboratory results showing worsening inflammation and organs dysfunction. AST: aspartate transaminase, ALT: alanine transaminase, ANC: absolute neutrophil count, WBC: white blood cells.

Lab	Patient's value on presentation	Patient's value on day 10	Patient’s value on day 16	Reference range
WBC	2.8 k/mm^3^	1.4 k/mm^3^	0.4 k/mm^3^	4.5–11.0 k/mm^3^
ANC	1.6 k/mm^3^	0.9 k/mm^3^	0.2 k/mm^3^	1.8–7.7 k/mm^3^
Hemoglobin	10 g/dL	9.4 g/dL	5.5 g/dL	13.5–17.5 g/dL
Platelets	138 k/mm^3^	66 k/mm^3^	11 k/mm^3^	150–400 k/mm^3^
Na	132 mmol\L	134 mmol\L	138 mmol\L	136–145 mmol\L
K	3.9 mmol\L	3.4 mmol\L	5.3 mmol\L	3.4–4.5 mmol\L
Cl	98 mmol\L	98 mmol\L	103 mmol\L	89–107 mmol\L
HCO_3_	28 mmol\L	27 mmol\L	<10 mmol\L	20–31 mmol\L
BUN	11 mg\dL	13 mg\dL	103 mg\dL	9–23 mg\dL
Cr	0.95 mg/dL	0.84 mg/dL	5.15 mg/dL	0.6–1.1 mg/dL
AST	53 units/L	291 units/L	3292 units/L	>33 units/L
ALT	33 units/L	165 units/L	1206 units/L	10–49 units/L
Ferritin	-	5133 ng/mL	>16,500 ng/mL	10.5–307.3 ng/mL
Triglyceride	-	251 mg/dL	-	<150 mg/dL
Fibrinogen	-	244 mg/dL	<60 mg/dL	195–505 mg/dL

**Figure 1:**
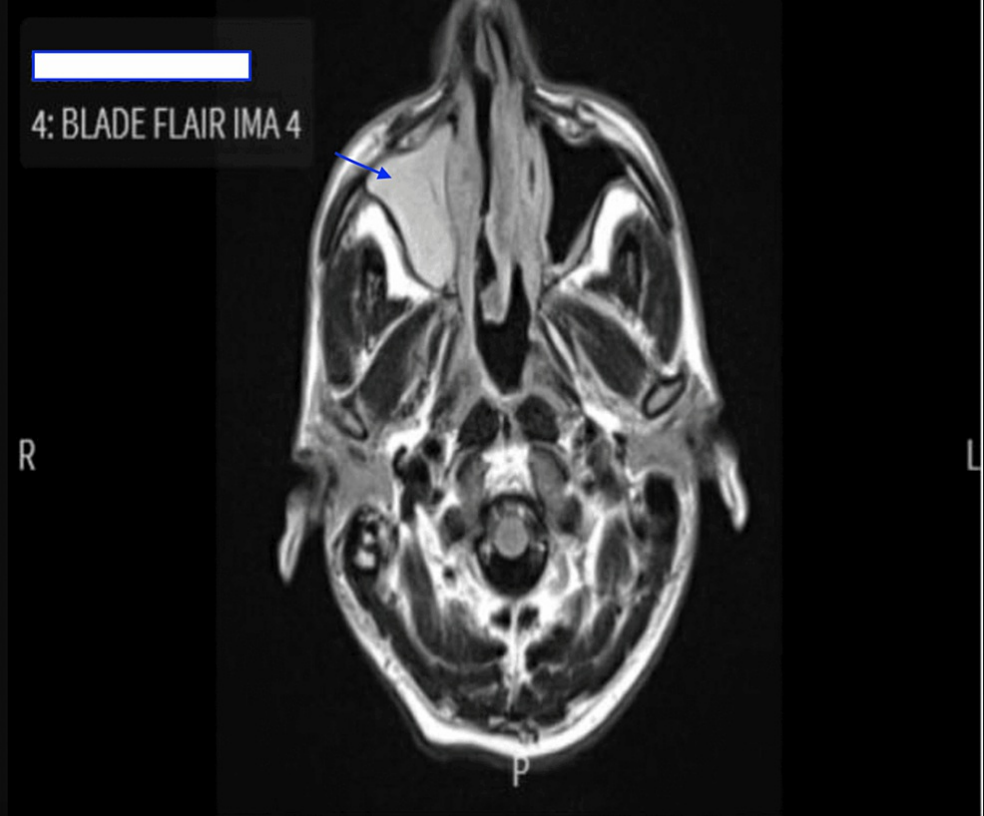
MRI brain with contrast showing necrosis of the soft tissues in the right nasal cavity.

A computed tomography (CT) maxillofacial scan with contrast showed complete opacification of the right frontal sinus and near-complete opacification of the right ethmoid and maxillary sinuses. In addition, magnetic resonance imaging (MRI) of the brain with contrast showed necrosis of the soft tissues in the right nasal cavity with no abnormalities in the brain (Figure 1). He was started on Piperacillin-Tazobactam for concerns of acute bacterial sinusitis but continued to have high-grade fevers (39.4 °C) and epistaxis, with notable splenomegaly on the exam. The patient’s laboratory results continued to worsen, showing organ dysfunction and worsening inflammation, as shown by the following. 

The workup for infectious sources, including blood and fungal cultures, human immunodeficiency virus (HIV), hepatitis panel, cytomegalovirus (CMV) PCR, and parvovirus B19, was negative. However, Epstein-Barr virus (EBV) PCR was found to be elevated at 9281 copies/mL. The workup for an underlying rheumatological disorder (anti-nuclear antibodies [ANA], rheumatoid factor [RF], dsDNA, and antineutrophil cytoplasmic antibody [ANCA]) was unremarkable.

**Figure 2:**
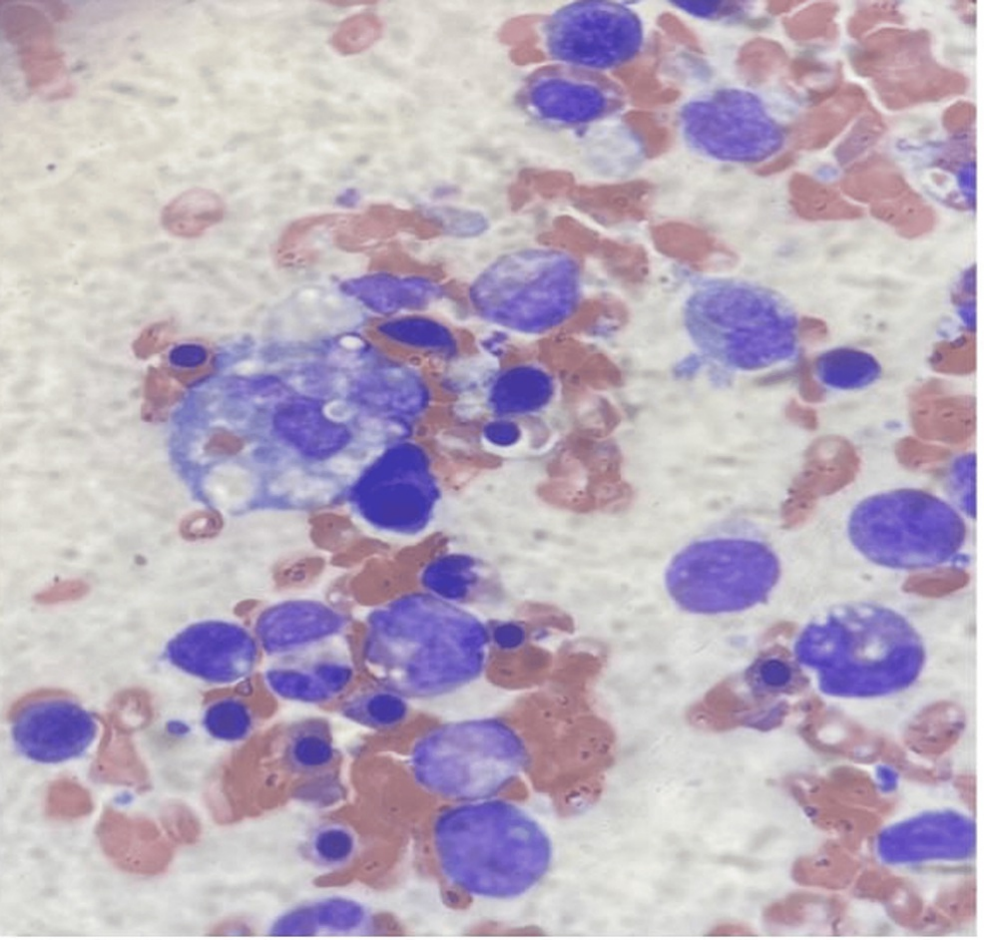
Hemophagocytes from bone marrow biopsy

**Figure 3:**
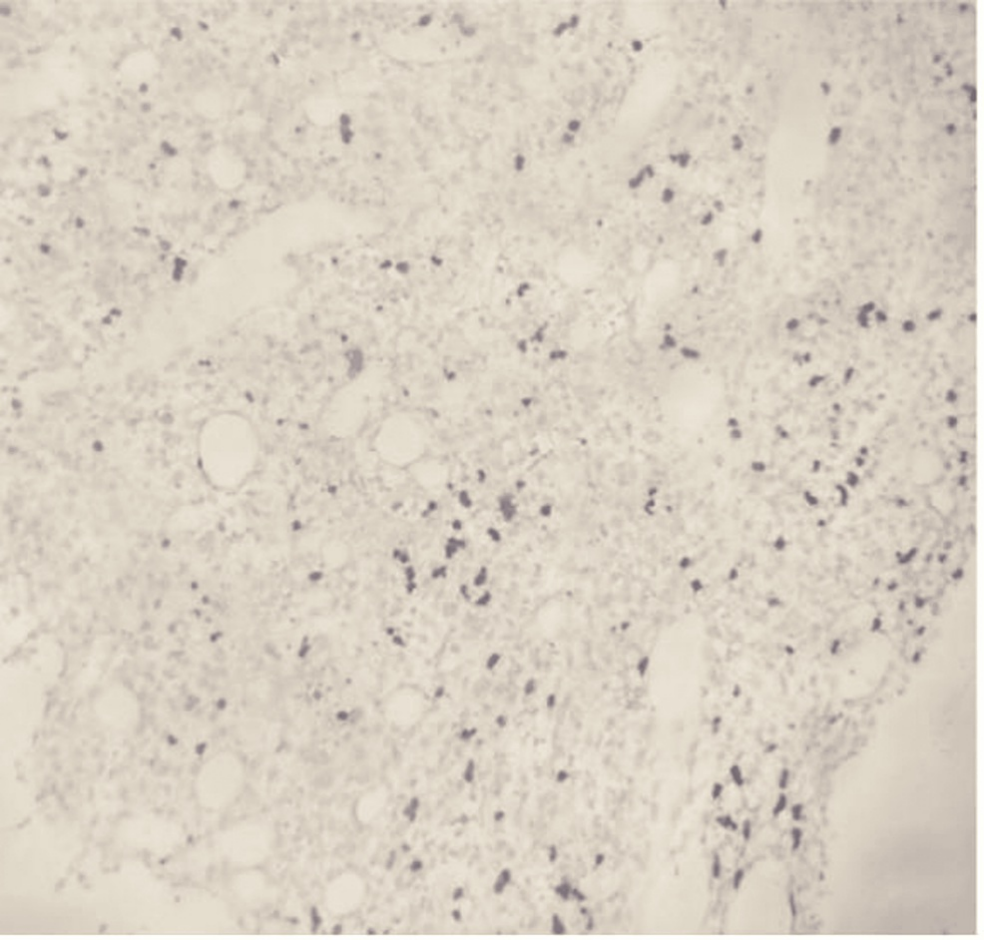
EBER probe stains on bone marrow biopsy, indicating EBV virus. EBER: Epstein-Barr virus (EBV)-encoded RNA.

He underwent a right nasal cavity biopsy, which revealed severe acute inflammation and bacterial colonies, however negative for fungal microorganisms or acid-fast bacilli. Flow cytometry showed no T-cell or B-cell lymphoid neoplasm or increase in circulating blasts. He also underwent a bone marrow biopsy in the interim, demonstrating EBV-associated marrow necrosis and atypical NK/T-cell proliferation with mild hemophagocytosis without immunophenotypic evidence of a monotypic population of B-cells, abnormal T-cells, or increased blasts. C-MYC rearrangement was negative. T-cell receptor Gamma gene rearrangement was negative, and T-cell receptor Beta gene rearrangement was indeterminate (Figures 2-3).

Given the above findings and elevated soluble Interleukin-2 (IL-2) receptor at 5483.2 pg/mL (normal range: 158-664 pg/mL), a clinical diagnosis of EBV-associated HLH was made (fever >38.5 °C, splenomegaly, pancytopenia, hypertriglyceridemia and hypofibrinogenemia, hemophagocytosis in bone marrow, ferritin >500, and elevated soluble IL-2 receptor), and the patient was started on pulse dose steroids (dexamethasone 15 mg) for three days. His condition and laboratory markers continued to deteriorate, with worsening multiorgan failure and a rising EBV viral load complicated by disseminated intravascular coagulation (DIC), raising suspicion for underlying extra-nodal NK/T cell lymphoma, which could not be confirmed as the patient’s severe coagulopathy limited him from getting further histopathological diagnosis. He was started on the HLH-94 protocol for treatment with etoposide and dexamethasone (10 mg\m^2^ daily). Ganciclovir was also added. Rituximab was also considered to reduce the EBV viral load. However, the patient had fast deterioration and succumbed to multiorgan failure after two days post-chemotherapy.

## Discussion

HLH is a rare, life-threatening syndrome that results from the uncontrolled activation of cytotoxic lymphocytes and macrophages. This activation, in turn, results in cytokine-mediated multiorgan dysfunction and tissue injury [1]. The exact mechanisms underlying HLH remain incompletely understood. HLH has an estimated incidence of 1.2 cases per million patients per year [3], with a 1:1 male-to-female ratio [1]. HLH has been historically divided into different forms, including primary, secondary, and reactive. Of note, the North American Consortium of Histiocytosis (NACHO) recommends a new classification system in which HLH is divided into HLH disease and HLH mimics based on the consensus diagnostic criteria. In all subtypes of HLH, higher cytokine levels are associated with a worse prognosis [1]. 

Secondary HLH is less common, especially in immunocompetent patients [3]. The secondary form of HLH could be due to malignancy, chronic inflammation, or, as in the case of our patient, infection. 

Infection-induced secondary HLH can be related to different pathogens such as EBV, HIV, influenza, mycobacterium, leishmania, histoplasmosis, and plasmodium. EBV-induced HLH is reported to account for roughly 40% of viral cases [4]. However, in their literature review, Koumadoraki et al. found that out of the 636 cases of HLH secondary to an infection, almost 50% were associated with an EBV infection [2]. What is unique to our patient is that his presenting symptom was epistaxis. EBV-infection-driven HLH occurs mostly in children and adolescents [1]. 

In adults, EBV-triggered HLH occurs mostly in the setting of immune compromise [1]. This adds to the peculiarity of our case, which presents a 55-year-old patient who had chronic sinusitis but was otherwise previously healthy. EBV-triggered HLH usually occurs in patients with X-linked lymphoproliferative disorder type 1 (XLP1), X-linked lymphoproliferative disorder type 2 (XLP2), IL-2-inducible T cell kinase deficiency, CD27 deficiency, and X-linked immunodeficiency with magnesium defect (XMEM) [1].

Patients with HLH usually present with elevated ferritin, fever, cytopenias, coagulopathy, organomegaly, lymphadenopathy, central nervous system dysfunction, and hepatic injury. These clinical findings usually progress rapidly, and patients deteriorate quickly. The H-score is used to determine the chance of having HLH. Our patient had an H-score of 199 points, which translates to a probability of 80-88% of having HLH. If left untreated, HLH is highly fatal in adults, with a mortality rate reaching as high as 75% [5]. Unfortunately, specific criteria for the diagnosis of secondary HLH are still being studied, as our current tools lack the necessary sensitivity to be clinically useful.

Treatment of HLH generally consists of controlling inflammation and addressing the underlying cause, such as an infection or malignancy. Treatment with the HLH-94 protocol consists of etoposide and dexamethasone, with the initial therapy lasting eight weeks (about two months). It has shown a possible cure in over half the patients, with 71% of patients reaching remission or transplant [6]. Unlike the primary/familiar form of HLH, where a transplant is the only recommended long-term curative treatment, in secondary HLH, a transplant needs to be assessed on a case-by-case basis, and a hematologist/oncologist should be involved early in the care [1]. Some of the recommended criteria for transplant include persistent NK cell dysfunction and CNS involvement [3]. Antiviral therapy and intravenous immunoglobulin can be considered on a case-by-case basis.

## Conclusions

Immune activation and subsequent cytokine storms from an infection are common triggers for HLH, both in patients with a genetic predisposition and in sporadic cases with no underlying genetic pathology. The most common infectious trigger is a viral infection, especially EBV. Our patient, who was immunocompetent, presented with a rapidly progressing viremia and HLH, leading to acute liver failure and DIC. A prompt diagnosis of HLH and its underlying culprit is crucial, given the high mortality associated with HLH.
